# Supplementary material for: Pyruvate Dehydrogenase Kinase 1 inhibition mediated oxidative phosphorylation enhancement in cartilage promotes osteoarthritis progression
Source: BMC Musculoskelet Disord. 2023 Jul 20;24:597. doi: 10.1186/s12891-023-06585-6 (PMC10357736; doi:10.1186/s12891-023-06585-6)
Supplement: Supplementary file 1 — Additional file 1. [file 12891_2023_6585_MOESM1_ESM.zip › BP.Gsea.1653623667859/GOBP_IMMUNOGLOBULIN_PRODUCTION.html]

Details for gene set GOBP\_IMMUNOGLOBULIN\_PRODUCTION[GSEA]

|  || Dataset | 55457\_collapsed\_to\_symbols.cls.cls #OA\_versus\_normal.cls.cls #OA\_versus\_normal\_repos |
| Phenotype | cls.cls#OA\_versus\_normal\_repos |
| Upregulated in class | OA |
| GeneSet | GOBP\_IMMUNOGLOBULIN\_PRODUCTION |
| Enrichment Score (ES) | 0.33746672 |
| Normalized Enrichment Score (NES) | 1.6115354 |
| Nominal p-value | 0.0 |
| FDR q-value | 0.24736202 |
| FWER p-Value | 1.0 |
Table: GSEA Results Summary

  

Fig 1: Enrichment plot: GOBP\_IMMUNOGLOBULIN\_PRODUCTION      
 Profile of the Running ES Score & Positions of GeneSet Members on the Rank Ordered List

  

| SYMBOL | TITLE | RANK IN GENE LIST | RANK METRIC SCORE | RUNNING ES | CORE ENRICHMENT || 1 | POLB | DNA polymerase beta [Source:HGNC Symbol;Acc:HGNC:9174] | 93 | 0.923 | 0.0180 | Yes |
| 2 | GPI | glucose-6-phosphate isomerase [Source:HGNC Symbol;Acc:HGNC:4458] | 166 | 0.842 | 0.0355 | Yes |
| 3 | TREX1 | three prime repair exonuclease 1 [Source:HGNC Symbol;Acc:HGNC:12269] | 393 | 0.709 | 0.0372 | Yes |
| 4 | MLH1 | mutL homolog 1 [Source:HGNC Symbol;Acc:HGNC:7127] | 497 | 0.660 | 0.0472 | Yes |
| 5 | IGKV1D-13 | immunoglobulin kappa variable 1D-13 [Source:HGNC Symbol;Acc:HGNC:5747] | 589 | 0.628 | 0.0573 | Yes |
| 6 | XRCC4 | X-ray repair cross complementing 4 [Source:HGNC Symbol;Acc:HGNC:12831] | 602 | 0.622 | 0.0734 | Yes |
| 7 | HLA-DMB | "major histocompatibility complex, class II, DM beta [Source:HGNC Symbol;Acc:HGNC:4935]" | 679 | 0.598 | 0.0839 | Yes |
| 8 | CD86 | CD86 molecule [Source:HGNC Symbol;Acc:HGNC:1705] | 689 | 0.596 | 0.0995 | Yes |
| 9 | IGKV1D-8 | immunoglobulin kappa variable 1D-8 [Source:HGNC Symbol;Acc:HGNC:5759] | 731 | 0.586 | 0.1124 | Yes |
| 10 | MSH2 | mutS homolog 2 [Source:HGNC Symbol;Acc:HGNC:7325] | 739 | 0.584 | 0.1278 | Yes |
| 11 | TNFSF13 | TNF superfamily member 13 [Source:HGNC Symbol;Acc:HGNC:11928] | 787 | 0.572 | 0.1398 | Yes |
| 12 | BTK | Bruton tyrosine kinase [Source:HGNC Symbol;Acc:HGNC:1133] | 889 | 0.545 | 0.1469 | Yes |
| 13 | PMS2P1 | "PMS1 homolog 2, mismatch repair system component pseudogene 1 [Source:HGNC Symbol;Acc:HGNC:9123]" | 917 | 0.537 | 0.1595 | Yes |
| 14 | IGKV1D-39 | immunoglobulin kappa variable 1D-39 [Source:HGNC Symbol;Acc:HGNC:5756] | 988 | 0.520 | 0.1683 | Yes |
| 15 | IGLV2-14 | immunoglobulin lambda variable 2-14 [Source:HGNC Symbol;Acc:HGNC:5888] | 1053 | 0.506 | 0.1771 | Yes |
| 16 | IGKV2D-28 | immunoglobulin kappa variable 2D-28 [Source:HGNC Symbol;Acc:HGNC:5799] | 1126 | 0.491 | 0.1849 | Yes |
| 17 | IGKV3-20 | immunoglobulin kappa variable 3-20 [Source:HGNC Symbol;Acc:HGNC:5817] | 1154 | 0.486 | 0.1961 | Yes |
| 18 | NDFIP1 | Nedd4 family interacting protein 1 [Source:HGNC Symbol;Acc:HGNC:17592] | 1183 | 0.480 | 0.2071 | Yes |
| 19 | IL21 | interleukin 21 [Source:HGNC Symbol;Acc:HGNC:6005] | 1199 | 0.477 | 0.2190 | Yes |
| 20 | IL13RA2 | interleukin 13 receptor subunit alpha 2 [Source:HGNC Symbol;Acc:HGNC:5975] | 1202 | 0.476 | 0.2319 | Yes |
| 21 | HLA-DQA1 | "major histocompatibility complex, class II, DQ alpha 1 [Source:HGNC Symbol;Acc:HGNC:4942]" | 1334 | 0.452 | 0.2340 | Yes |
| 22 | IGKC | immunoglobulin kappa constant [Source:HGNC Symbol;Acc:HGNC:5716] | 1477 | 0.432 | 0.2347 | Yes |
| 23 | HLA-DQB2 | "major histocompatibility complex, class II, DQ beta 2 [Source:HGNC Symbol;Acc:HGNC:4945]" | 1534 | 0.423 | 0.2419 | Yes |
| 24 | CYREN | cell cycle regulator of NHEJ [Source:HGNC Symbol;Acc:HGNC:22432] | 1635 | 0.408 | 0.2453 | Yes |
| 25 | HLA-DMA | "major histocompatibility complex, class II, DM alpha [Source:HGNC Symbol;Acc:HGNC:4934]" | 1769 | 0.388 | 0.2455 | Yes |
| 26 | AICDA | activation induced cytidine deaminase [Source:HGNC Symbol;Acc:HGNC:13203] | 1825 | 0.381 | 0.2517 | Yes |
| 27 | IGKV1D-17 | immunoglobulin kappa variable 1D-17 [Source:HGNC Symbol;Acc:HGNC:5749] | 1859 | 0.378 | 0.2595 | Yes |
| 28 | TRAF3IP2 | TRAF3 interacting protein 2 [Source:HGNC Symbol;Acc:HGNC:1343] | 1939 | 0.368 | 0.2634 | Yes |
| 29 | IGLV3-25 | immunoglobulin lambda variable 3-25 [Source:HGNC Symbol;Acc:HGNC:5908] | 1977 | 0.363 | 0.2704 | Yes |
| 30 | FCGR2B | Fc fragment of IgG receptor IIb [Source:HGNC Symbol;Acc:HGNC:3618] | 2016 | 0.360 | 0.2773 | Yes |
| 31 | IGLV3-10 | immunoglobulin lambda variable 3-10 [Source:HGNC Symbol;Acc:HGNC:5897] | 2047 | 0.356 | 0.2847 | Yes |
| 32 | CCR6 | C-C motif chemokine receptor 6 [Source:HGNC Symbol;Acc:HGNC:1607] | 2095 | 0.350 | 0.2906 | Yes |
| 33 | TNF | tumor necrosis factor [Source:HGNC Symbol;Acc:HGNC:11892] | 2098 | 0.350 | 0.3001 | Yes |
| 34 | MZB1 | marginal zone B and B1 cell specific protein [Source:HGNC Symbol;Acc:HGNC:30125] | 2123 | 0.347 | 0.3077 | Yes |
| 35 | IGKV4-1 | immunoglobulin kappa variable 4-1 [Source:HGNC Symbol;Acc:HGNC:5834] | 2132 | 0.346 | 0.3166 | Yes |
| 36 | HLA-DOB | "major histocompatibility complex, class II, DO beta [Source:HGNC Symbol;Acc:HGNC:4937]" | 2147 | 0.344 | 0.3249 | Yes |
| 37 | VPREB3 | V-set pre-B cell surrogate light chain 3 [Source:HGNC Symbol;Acc:HGNC:12710] | 2246 | 0.333 | 0.3264 | Yes |
| 38 | SASH3 | SAM and SH3 domain containing 3 [Source:HGNC Symbol;Acc:HGNC:15975] | 2335 | 0.322 | 0.3283 | Yes |
| 39 | IGLV3-19 | immunoglobulin lambda variable 3-19 [Source:HGNC Symbol;Acc:HGNC:5903] | 2440 | 0.309 | 0.3286 | Yes |
| 40 | TGFB1 | transforming growth factor beta 1 [Source:HGNC Symbol;Acc:HGNC:11766] | 2490 | 0.303 | 0.3331 | Yes |
| 41 | PKN1 | protein kinase N1 [Source:HGNC Symbol;Acc:HGNC:9405] | 2540 | 0.298 | 0.3375 | Yes |
| 42 | LIG4 | DNA ligase 4 [Source:HGNC Symbol;Acc:HGNC:6601] | 2668 | 0.283 | 0.3353 | No |
| 43 | HLA-DQB1 | "major histocompatibility complex, class II, DQ beta 1 [Source:HGNC Symbol;Acc:HGNC:4944]" | 2840 | 0.266 | 0.3292 | No |
| 44 | ERCC1 | "ERCC excision repair 1, endonuclease non-catalytic subunit [Source:HGNC Symbol;Acc:HGNC:3433]" | 2948 | 0.255 | 0.3278 | No |
| 45 | IL27RA | interleukin 27 receptor subunit alpha [Source:HGNC Symbol;Acc:HGNC:17290] | 3298 | 0.221 | 0.3066 | No |
| 46 | SHLD2 | shieldin complex subunit 2 [Source:HGNC Symbol;Acc:HGNC:28773] | 3300 | 0.221 | 0.3125 | No |
| 47 | EPHB2 | EPH receptor B2 [Source:HGNC Symbol;Acc:HGNC:3393] | 3677 | 0.188 | 0.2883 | No |
| 48 | XCL1 | X-C motif chemokine ligand 1 [Source:HGNC Symbol;Acc:HGNC:10645] | 3789 | 0.178 | 0.2845 | No |
| 49 | TMBIM6 | transmembrane BAX inhibitor motif containing 6 [Source:HGNC Symbol;Acc:HGNC:11723] | 4045 | 0.152 | 0.2687 | No |
| 50 | NBN | nibrin [Source:HGNC Symbol;Acc:HGNC:7652] | 4136 | 0.143 | 0.2655 | No |
| 51 | POLQ | DNA polymerase theta [Source:HGNC Symbol;Acc:HGNC:9186] | 4293 | 0.129 | 0.2569 | No |
| 52 | CD22 | CD22 molecule [Source:HGNC Symbol;Acc:HGNC:1643] | 4296 | 0.128 | 0.2602 | No |
| 53 | HLA-DOA | "major histocompatibility complex, class II, DO alpha [Source:HGNC Symbol;Acc:HGNC:4936]" | 4344 | 0.125 | 0.2600 | No |
| 54 | PHB2 | prohibitin 2 [Source:HGNC Symbol;Acc:HGNC:30306] | 4354 | 0.124 | 0.2626 | No |
| 55 | TNFRSF4 | TNF receptor superfamily member 4 [Source:HGNC Symbol;Acc:HGNC:11918] | 4383 | 0.122 | 0.2638 | No |
| 56 | CD28 | CD28 molecule [Source:HGNC Symbol;Acc:HGNC:1653] | 4543 | 0.110 | 0.2544 | No |
| 57 | PMS2P5 | "PMS1 homolog 2, mismatch repair system component pseudogene 5 [Source:HGNC Symbol;Acc:HGNC:9130]" | 4623 | 0.103 | 0.2510 | No |
| 58 | RNF8 | ring finger protein 8 [Source:HGNC Symbol;Acc:HGNC:10071] | 4938 | 0.077 | 0.2286 | No |
| 59 | TP53BP1 | tumor protein p53 binding protein 1 [Source:HGNC Symbol;Acc:HGNC:11999] | 4946 | 0.076 | 0.2301 | No |
| 60 | ATAD5 | ATPase family AAA domain containing 5 [Source:HGNC Symbol;Acc:HGNC:25752] | 5020 | 0.071 | 0.2263 | No |
| 61 | VAMP3 | vesicle associated membrane protein 3 [Source:HGNC Symbol;Acc:HGNC:12644] | 5028 | 0.071 | 0.2277 | No |
| 62 | RBP4 | retinol binding protein 4 [Source:HGNC Symbol;Acc:HGNC:9922] | 5072 | 0.067 | 0.2262 | No |
| 63 | PHB | prohibitin [Source:HGNC Symbol;Acc:HGNC:8912] | 5102 | 0.064 | 0.2257 | No |
| 64 | TRAV9-2 | T cell receptor alpha variable 9-2 [Source:HGNC Symbol;Acc:HGNC:12154] | 5105 | 0.064 | 0.2273 | No |
| 65 | NHEJ1 | non-homologous end joining factor 1 [Source:HGNC Symbol;Acc:HGNC:25737] | 5150 | 0.060 | 0.2255 | No |
| 66 | IL5 | interleukin 5 [Source:HGNC Symbol;Acc:HGNC:6016] | 5277 | 0.050 | 0.2170 | No |
| 67 | HMCES | "5-hydroxymethylcytosine binding, ES cell specific [Source:HGNC Symbol;Acc:HGNC:24446]" | 5402 | 0.040 | 0.2084 | No |
| 68 | HLA-DPB1 | "major histocompatibility complex, class II, DP beta 1 [Source:HGNC Symbol;Acc:HGNC:4940]" | 5486 | 0.033 | 0.2028 | No |
| 69 | CD40LG | CD40 ligand [Source:HGNC Symbol;Acc:HGNC:11935] | 5529 | 0.030 | 0.2003 | No |
| 70 | TRAF6 | TNF receptor associated factor 6 [Source:HGNC Symbol;Acc:HGNC:12036] | 5761 | 0.011 | 0.1826 | No |
| 71 | SAMHD1 | SAM and HD domain containing deoxynucleoside triphosphate triphosphohydrolase 1 [Source:HGNC Symbol;Acc:HGNC:15925] | 5790 | 0.009 | 0.1806 | No |
| 72 | IL2 | interleukin 2 [Source:HGNC Symbol;Acc:HGNC:6001] | 5819 | 0.007 | 0.1786 | No |
| 73 | TNFSF4 | TNF superfamily member 4 [Source:HGNC Symbol;Acc:HGNC:11934] | 5930 | 0.000 | 0.1700 | No |
| 74 | DNAJB9 | DnaJ heat shock protein family (Hsp40) member B9 [Source:HGNC Symbol;Acc:HGNC:6968] | 5936 | -0.000 | 0.1696 | No |
| 75 | UNG | uracil DNA glycosylase [Source:HGNC Symbol;Acc:HGNC:12572] | 6078 | -0.011 | 0.1589 | No |
| 76 | KMT5B | lysine methyltransferase 5B [Source:HGNC Symbol;Acc:HGNC:24283] | 6198 | -0.020 | 0.1501 | No |
| 77 | POLL | DNA polymerase lambda [Source:HGNC Symbol;Acc:HGNC:9184] | 6214 | -0.022 | 0.1495 | No |
| 78 | CTNNBL1 | catenin beta like 1 [Source:HGNC Symbol;Acc:HGNC:15879] | 6493 | -0.042 | 0.1289 | No |
| 79 | HLA-DRA | "major histocompatibility complex, class II, DR alpha [Source:HGNC Symbol;Acc:HGNC:4947]" | 6550 | -0.046 | 0.1258 | No |
| 80 | IL4 | interleukin 4 [Source:HGNC Symbol;Acc:HGNC:6014] | 6673 | -0.054 | 0.1177 | No |
| 81 | CD40 | CD40 molecule [Source:HGNC Symbol;Acc:HGNC:11919] | 6825 | -0.065 | 0.1077 | No |
| 82 | FOXP3 | forkhead box P3 [Source:HGNC Symbol;Acc:HGNC:6106] | 7035 | -0.082 | 0.0936 | No |
| 83 | HLA-DRB1 | "major histocompatibility complex, class II, DR beta 1 [Source:HGNC Symbol;Acc:HGNC:4948]" | 7087 | -0.086 | 0.0920 | No |
| 84 | IGLV4-60 | immunoglobulin lambda variable 4-60 [Source:HGNC Symbol;Acc:HGNC:5920] | 7108 | -0.089 | 0.0928 | No |
| 85 | TBX21 | T-box transcription factor 21 [Source:HGNC Symbol;Acc:HGNC:11599] | 7207 | -0.097 | 0.0878 | No |
| 86 | RIF1 | replication timing regulatory factor 1 [Source:HGNC Symbol;Acc:HGNC:23207] | 7448 | -0.114 | 0.0722 | No |
| 87 | TCF3 | transcription factor 3 [Source:HGNC Symbol;Acc:HGNC:11633] | 7521 | -0.120 | 0.0698 | No |
| 88 | NSD2 | nuclear receptor binding SET domain protein 2 [Source:HGNC Symbol;Acc:HGNC:12766] | 7622 | -0.129 | 0.0655 | No |
| 89 | PTPRC | protein tyrosine phosphatase receptor type C [Source:HGNC Symbol;Acc:HGNC:9666] | 7744 | -0.137 | 0.0598 | No |
| 90 | MCM3AP | minichromosome maintenance complex component 3 associated protein [Source:HGNC Symbol;Acc:HGNC:6946] | 8077 | -0.161 | 0.0383 | No |
| 91 | EXO1 | exonuclease 1 [Source:HGNC Symbol;Acc:HGNC:3511] | 8088 | -0.162 | 0.0419 | No |
| 92 | PAXIP1 | PAX interacting protein 1 [Source:HGNC Symbol;Acc:HGNC:8624] | 8148 | -0.167 | 0.0419 | No |
| 93 | PMS2 | "PMS1 homolog 2, mismatch repair system component [Source:HGNC Symbol;Acc:HGNC:9122]" | 8172 | -0.169 | 0.0447 | No |
| 94 | STAT6 | signal transducer and activator of transcription 6 [Source:HGNC Symbol;Acc:HGNC:11368] | 8191 | -0.170 | 0.0480 | No |
| 95 | TRAF2 | TNF receptor associated factor 2 [Source:HGNC Symbol;Acc:HGNC:12032] | 8322 | -0.180 | 0.0427 | No |
| 96 | VPREB1 | V-set pre-B cell surrogate light chain 1 [Source:HGNC Symbol;Acc:HGNC:12709] | 8339 | -0.181 | 0.0464 | No |
| 97 | SUPT6H | "SPT6 homolog, histone chaperone and transcription elongation factor [Source:HGNC Symbol;Acc:HGNC:11470]" | 8752 | -0.213 | 0.0200 | No |
| 98 | MSH6 | mutS homolog 6 [Source:HGNC Symbol;Acc:HGNC:7329] | 8846 | -0.221 | 0.0188 | No |
| 99 | IL10 | interleukin 10 [Source:HGNC Symbol;Acc:HGNC:5962] | 8855 | -0.221 | 0.0242 | No |
| 100 | PARP3 | poly(ADP-ribose) polymerase family member 3 [Source:HGNC Symbol;Acc:HGNC:273] | 9186 | -0.246 | 0.0052 | No |
| 101 | STX4 | syntaxin 4 [Source:HGNC Symbol;Acc:HGNC:11439] | 9222 | -0.250 | 0.0093 | No |
| 102 | HLA-DPA1 | "major histocompatibility complex, class II, DP alpha 1 [Source:HGNC Symbol;Acc:HGNC:4938]" | 9481 | -0.273 | -0.0034 | No |
| 103 | PRKDC | "protein kinase, DNA-activated, catalytic subunit [Source:HGNC Symbol;Acc:HGNC:9413]" | 9632 | -0.286 | -0.0073 | No |
| 104 | MSH3 | mutS homolog 3 [Source:HGNC Symbol;Acc:HGNC:7326] | 9796 | -0.301 | -0.0118 | No |
| 105 | SWAP70 | switching B cell complex subunit SWAP70 [Source:HGNC Symbol;Acc:HGNC:17070] | 10019 | -0.323 | -0.0203 | No |
| 106 | TFRC | transferrin receptor [Source:HGNC Symbol;Acc:HGNC:11763] | 10184 | -0.339 | -0.0239 | No |
| 107 | BATF | basic leucine zipper ATF-like transcription factor [Source:HGNC Symbol;Acc:HGNC:958] | 10312 | -0.352 | -0.0242 | No |
| 108 | IL4R | interleukin 4 receptor [Source:HGNC Symbol;Acc:HGNC:6015] | 10902 | -0.417 | -0.0588 | No |
| 109 | HPX | hemopexin [Source:HGNC Symbol;Acc:HGNC:5171] | 11156 | -0.451 | -0.0663 | No |
| 110 | CR1 | complement C3b/C4b receptor 1 (Knops blood group) [Source:HGNC Symbol;Acc:HGNC:2334] | 11376 | -0.483 | -0.0702 | No |
| 111 | HSPD1 | heat shock protein family D (Hsp60) member 1 [Source:HGNC Symbol;Acc:HGNC:5261] | 11427 | -0.492 | -0.0606 | No |
| 112 | HLA-E | "major histocompatibility complex, class I, E [Source:HGNC Symbol;Acc:HGNC:4962]" | 11659 | -0.526 | -0.0643 | No |
| 113 | IL13 | interleukin 13 [Source:HGNC Symbol;Acc:HGNC:5973] | 12173 | -0.636 | -0.0870 | No |
| 114 | YY1 | YY1 transcription factor [Source:HGNC Symbol;Acc:HGNC:12856] | 12229 | -0.653 | -0.0734 | No |
| 115 | CLCF1 | cardiotrophin like cytokine factor 1 [Source:HGNC Symbol;Acc:HGNC:17412] | 12360 | -0.695 | -0.0645 | No |
| 116 | IL6 | interleukin 6 [Source:HGNC Symbol;Acc:HGNC:6018] | 12460 | -0.730 | -0.0522 | No |
| 117 | THOC1 | THO complex 1 [Source:HGNC Symbol;Acc:HGNC:19070] | 12503 | -0.749 | -0.0350 | No |
| 118 | B2M | beta-2-microglobulin [Source:HGNC Symbol;Acc:HGNC:914] | 12507 | -0.750 | -0.0147 | No |
| 119 | HLA-DRB4 | "major histocompatibility complex, class II, DR beta 4 [Source:HGNC Symbol;Acc:HGNC:4952]" | 12636 | -0.815 | -0.0024 | No |
| 120 | BCL6 | BCL6 transcription repressor [Source:HGNC Symbol;Acc:HGNC:1001] | 12667 | -0.838 | 0.0182 | No |
Table: GSEA details [plain text format]

  

Fig 2: GOBP\_IMMUNOGLOBULIN\_PRODUCTION      
 Blue-Pink O' Gram in the Space of the Analyzed GeneSet

  

Fig 3: GOBP\_IMMUNOGLOBULIN\_PRODUCTION: Random ES distribution      
 Gene set null distribution of ES for **GOBP\_IMMUNOGLOBULIN\_PRODUCTION**
